# Neighborhood environment correlates of physical activity and sedentary behavior among Latino adults in Massachusetts

**DOI:** 10.1186/s12889-016-3650-4

**Published:** 2016-09-13

**Authors:** Valerie J. Silfee, Milagros C. Rosal, Meera Sreedhara, Vilma Lora, Stephenie C. Lemon

**Affiliations:** 1 Division of Preventive and Behavioral Medicine, Department of Medicine, University of Massachusetts Medical School, 55 Lake Avenue North, Worcester, MA 01655 USA; 2 City of Lawrence Mayor’s Health Task Force and YWCA of Greater Lawrence, Lawrence, MA USA

**Keywords:** Cardiovascular disease, Physical activity, Sedentary behavior, Neighborhood environment, Latino, Hispanic

## Abstract

**Background:**

U.S. Latinos experience high rates of cardio-metabolic diseases and have high rates of physical inactivity and sedentary behavior. Understanding the environmental factors associated with physical activity and sedentary behaviors among Latinos could inform future interventions. The purpose of this study is to explore the neighborhood environment correlates of physical activity and sedentary behavior in a sample of U.S. Latino adults.

**Methods:**

Cross-sectional study of 602 Latino adults in Lawrence, MA. Survey assessments of physical activity, sedentary behavior, and neighborhood environment were verbally administered. The neighborhood environment scale assessed violence, safety, aesthetic quality, walkability, availability of healthy foods, social cohesion, and activities with neighbors.

**Results:**

After controlling forage, gender, education, body mass index (BMI), and smoking status, two variables were associated with the outcomes of interest. Living in more walkable neighborhoods was associated with an increased likelihood of engaging in adequate levels of physical activity (>150 min per week, as recommended by the American College of Sports Medicine (ACSM)) (OR = 1.403, *p* = .018); and greater frequency of activities with neighbors was associated with greater sedentary behavior (β = .072, *p* = .05).

**Conclusions:**

There were different neighborhood environment correlates of physical activity and sedentary behavior in this Latino community. Focusing on a greater understanding of the distinct social and physical environmental correlates of physical activity and sedentary behavior may provide important insights for reducing CVD risk and health disparities among Latinos.

## Background

Latinos in the United States experience disproportionately high rates of obesity, type 2 diabetes and cardiovascular disease (CVD) [1, 2]. Less than one third (32.6 %) of White adults are obese compared to 42.5 % of Latino adults [3]. U.S. Latinos are also two-thirds more likely to develop type 2 diabetes, with a prevalence rate of 16.9 % versus 10.2 % among non-Hispanic Whites [4]. Additionally, as of 2013, approximately one-third of adult U.S. Latino men (33.4 %) and women (30.7 %) aged 20 years and older have CVD [2, 5].

Both sedentary behavior and physical activity have been consistently identified as important for cardio-metabolic disease prevention, including lower risk for obesity, diabetes, CVD, and all-cause mortality [1, 6]. However, currently, 59.8 % of U.S. Latino adults do not meet the American College of Sports Medicine (ACSM) recommended guidelines for physical activity (>150 min per week) [4]. It is also estimated that the general adult population spends up to 70 % of their waking hours in sedentary activities such as watching TV, using a computer, sitting at work, and transportation [7–9]. Emerging research also suggests that Latinos also may be spending up to 74 % of their waking hours being sedentary [10].

Environmental factors such as individuals’ neighborhoods may also increase risk for CVD, as communities with lower socioeconomic status (SES), such as those in which U.S. Latinos often live, have higher rates of morbidity and mortality [11–13]. However, much of the literature on the influence of the neighborhood environment on CVD uses U.S. Census Bureau data, limiting our ability to investigate specific neighborhood attributes relevant to individual health behaviors such as physical activity and sedentary behavior [12]. As a result, researchers and public health practitioners need to have a better understanding of specific aspects of the environment associated with health behaviors in order to develop more comprehensive CVD prevention interventions [14].

While studies have found neighborhood characteristics such as physical activity facilities, walkability (i.e. measure of how conducive an area is to walking), aesthetics, and safety are associated with physical activity in the general population [15–19], the relationship among U.S. Latinos remains unclear. Two studies among Latina women and overweight and obese African American and Latina women found no associations between self-reported or accelerometer- measured physical activity and selected neighborhood environmental factors such as access to physical activity resources (measured via proximity, availability, and cost), neighborhood safety from crime, neighborhood cohesion, and the pedestrian environment (i.e. availability of sidewalks) [20–22]. These studies counter findings in Latina immigrants which negatively associated physical activity and access to indoor and outdoor places for exercise, heavy traffic, speedy drivers, and unattended dogs in the neighborhood [23]. Together, these studies represent a body of research limited by inconsistent environmental factors, sole focus on Latina women, and inclusion of other minority populations; highlighting the need to further examine the relationship between physical activity and factors of the neighborhood environment where U.S. Latinos live.

Several studies have examined the relationship between sedentary behavior and neighborhood environment. A recent review, of studies among primarily with white populations, has reported mixed results between neighborhood environment variables and selected sedentary behaviors/sedentary time [24]. Sedentary activities such as TV viewing and more time spent sitting in a car have been associated with lower levels of neighborhood walkability [25]. However, living in medium-and high-walkable areas was associated with less TV watching in a sample of Australian women [26]. Finally, although driving longer distances has been related to living in low-walkable suburban neighborhoods [27], neighborhood walkability was associated with higher levels of sedentary time among Belgian adults [28]. To our knowledge, only one study [29] has examined the relationship between neighborhood environment and sedentary behavior and in Latinos; positively associating neighborhood attractiveness and safety with more time sitting in a car, but not overall sedentary time [22, 29].

These uncertain associations between neighborhood environment factors and sedentary behavior and physical activity warrant further investigation, particularly among U.S. Latinos, a population with considerable CVD risk. Having a greater understanding of the association between neighborhood environment attributes and physical activity and sedentary behaviors may provide insights for public health interventions seeking to reduce CVD risk and health disparities. Additionally, given the differences between physical activity and sedentariness [9], it is likely that these behaviors have unique sets of environmental determinants [25]. Therefore, the purpose of this study was to explore the association between selected neighborhood environment factors and physical activity and sedentary behavior in a sample of Latino adults.

## Methods

### Study design

This is a cross-sectional analysis of the Lawrence Health and Well Being Study of Latino adults in the city of Lawrence, Massachusetts. Participants were patients at the Greater Lawrence Family Health Center (GLFHC), a federally qualified community health center that sees an estimated 80–85 % of the Lawrence area Latino population. Proportional sampling within specified age (21–34, 35–54, 55–85) and gender strata, using electronic patient records, randomly selected potential research participants who met inclusion and exclusion criteria. Inclusion criteria included, being of Latino or Hispanic ethnicity, Spanish or English speaking, and between 21 and 85 years of age. Ethnicity was self-reported, and we included individuals who perceived themselves at Hispanic or Latinos. Of note, a majority of the sample (73.4 %) reported being of Dominican ethnicity. Individuals were excluded if they were unable or unwilling to give informed consent, planned to move out of the area within the original study period, had cognitive impairments that precluded participation (i.e. answering verbally administered questions), and/or had a life expectancy of less than 5 years, as determined by their primary care provider (PCP). Letters signed by the chief medical officer that included a description of the study (in Spanish and English) were sent to eligible participants. The letters stated that a study coordinator would call patients to provide additional information about the study, assess eligibility, and inquire about interest in participating. A toll-free number was provided for patients to call if they did not wish to participate. Bilingual/bicultural coordinators contacted patients and patients who were interested and eligible were invited to participate. Each individual assessment took place at a community site with easy access to participants (the Lawrence Senior center), were administered verbally by trained staff, and lasted approximately 2.5–3 h.

### Measures

#### Demographic characteristics

Individuals self-reported their gender, age, employment, education, and smoking status. Smoking status was assessed by asking individuals if they currently smoke every day, some days, or not at all. BMI was calculated from height and weight measured by study staff.

#### Physical activity

Physical activity was measured using the Women’s Health Initiative (WHI) Brief Physical Activity Questionnaire [30]. This 9-item multiple choice questionnaire assessed leisure-time walking as well as mild, moderate, and vigorous physical activity. For each activity category, individuals were asked to report frequency (“How many days of the week do you usually do mild exercise?”) and duration (“When you exercise like this, how long do you do it for?”). Frequency was assessed with a 5-point scale ranging from “never” to “7 + times” per week for walking, and ranging from “1 day” to “5 or more days” for mild, moderate, and vigorous exercise. Duration was assessed on a 4-point scale ranging from “less than 20 min” to “1 h or more”. Physical activity per week was obtained by multiplying frequency and duration multiple choice categories. Categories with a range (i.e. 20–39 min) were recoded to their midpoint values [30]. Due to the high proportion of individuals participating in 0 min of physical activity per week, the data was further coded to categorize individuals as engaging in 0 min of physical activity per week (inactive), >0–150 min of physical activity per week (not meeting guidelines), or >150 min per week (meeting guidelines). This questionnaire has been validated with adequate sensitivity and measurement bias compared to widely accepted physical activity measures [30].

#### Sedentary behavior

Sedentary behavior was assessed via The Sedentary Behavior Questionnaire [31]. Using this 22-item measure, individuals reported the amount of time they spent engaging in a set of sedentary behaviors on a scale 0 (none) to 9 (6 or more hours). Sedentary behaviors include sitting while: watching television, playing computer/video games, using the computer or Internet, listening to music, talking on the phone, doing paperwork or office work, reading, playing a musical instrument, doing arts and crafts, and driving or riding in a car, bus, or train. We modified the original scale to add additional sedentary activities: computer time and texting while sitting. For each behavior, individuals report average duration per day on weekdays and weekend days separately. Total sedentary time was calculated by converting scale ratings to number of hours. Weekday hours were multiplied by 5, weekend hours were multiplied by 2, and sums for total hours/week were averaged across 7 days. This questionnaire has a high intraclass correlation via test-retest reliability, and has modest associations with objective measures of sitting [31].

#### Environment

The Mujahid Neighborhood Health Questionnaire measured seven social and physical neighborhood environment features that may be associated with CVD risk, namely violence, safety, aesthetic quality, walking environment, availability of healthy foods, social cohesion, and activities with neighbors [11]. For safety, aesthetic quality, walking environment, availability of healthy foods, and social cohesion, responses were given on a 5-point Likert scale ranging from “strongly disagree” to “strongly agree”. Scales for violence and activities with neighbors ranged from 1 (often) to 4 (never). Scores were estimated by averaging all items within the scale. Scores were reversed in order to improve clarity of interpretation so that higher scores suggested higher neighborhood violence, safety, aesthetic quality, walkability, available healthy foods, social cohesion, and activities with neighbors.. This has shown to be a valid and reliable measure, with Cronbach’s alphas ranging from 0.73 to 0.83 and test-retest reliabilities of 0.6 to 0.88, and reliabilities of greater than 0.64 and 0.78 for objectively measured census tracts and census clusters, respectively [11].

### Statistical analysis

SPSS IBM Statistics (version 23) software was used for data analysis. Means, standard deviations, and frequencies were calculated for demographic variables as well as neighborhood variables, physical activity, and sedentary behavior. Multinomial logistic regression assessed the association of perceived neighborhood violence, safety, aesthetic quality, walking environment, availability of healthy foods, social cohesion, and activities with neighbors with level of physical activity engagement. Multiple linear regression models evaluated the extent to which perceived neighborhood violence, safety, aesthetic quality, walking environment, availability of healthy foods, social cohesion, and activities with neighbors were associated with total time spent in sedentary behavior. Perceived neighborhood variables were examined separately due to collinearity between subscales, as suggested by the survey developers [32]. Each perceived neighborhood variables was entered first into an unadjusted model for each outcome, and then a model adjusted for age, gender, BMI, education level, and smoking.

## Results

### Sample characteristics

A total of 602 Latino adults participated in this study. Demographic characteristics for the study participants are presented in Table 1. The mean age was 46.64 years (*SD* = 15.45) and mean BMI was 29.79 kg/m^2^ (*SD* = 5.97). Half (51.2 %) of the sample was female, over half (59.3 %) was employed, a majority were married or living with a partner (57.1 %), and almost one-third had at least come college (17.8 %) or a college degree (11.1 %). Less than half (41.7 %) met the physical activity guidelines of at least 150 min per week and 39.9 % of the sample participated in 0 min of physical activity per week. Participants engaged in an average 7.32 h of sedentary time per day (*SD* = 4.8). Means and standard deviations for neighborhood environment attributes are presented in Table 2. Values were calculated as averages from the given subscales and either scaled from 1 to 4 or 1 to 5.

**Table 1:** Demographic characteristics of participants in the Lawrence Health and Well-being Study, 2011–2013

Variable	Mean	*SD*
Age	46.64	15.45
BMI	29.79	5.97
Sedentary time (hours/day)	7.32	4.80
Variable	*n*	Frequency (%)
*Gender*		
Male	294	48.8
Female	308	51.2
*Employment*		
Employed	357	59.3
Unemployed	99	16.4
Retired	57	9.5
Disabled	56	9.3
Other	31	5.1
*Education*		
< High School	252	41.9
< High School, w/vocational training	56	9.3
High school graduate	67	11.1
High school graduate, w/vocational training	53	8.8
Some college	107	17.8
College degree or Post-Graduate	67	11.1
*Marital Status*		
Single (never married)	119	19.8
Married or Living with partner	344	57.1
Previously married (separated, divorced, widowed)	139	23.1
*Physical Activity*		
No Activity	240	39.9
> 0 and <150 min per Week	111	18.4
> 150 min per Week	251	41.7

**Table 2:** Means and standard deviations for Neighborhood Environmental Attributes in the Lawrence Health and Well-being Study, 2011–2013

Neighborhood attribute	Mean	*SD*
Violence^a^	1.52	0.69
Safety^b^	3.23	0.93
Aesthetic quality^b^	3.40	0.83
Walkability^b^	3.63	0.68
Availability of healthy foods^b^	3.15	1.06
Social cohesion^b^	3.16	0.81
Activities with neighbors^a^	2.11	0.88

Note: ^a^Scored on a scale from 1 to 4

^b^Scored on a scale from 1 to 5

Higher scored indicate higher violence, safety, aesthetic quality, walkability, availability of healthy foods, social cohesion, activities with neighbors

### Neighborhood environment and physical activity

Table 3 presents the results of the series of multinomial logistic regression models evaluating the associations between neighborhood characteristics, including perceived neighborhood violence, safety, aesthetic quality, walking environment, availability of healthy foods, social cohesion and activities with neighbors, and physical activity. In the unadjusted analyses, individuals who perceived their neighborhood as more walkable (OR = 1.445, *p* = .007) and to have higher availability of healthy foods (OR = 1.201, *p* = .033) were more likely to engage in more than 150 min of physical activity per week. After controlling for demographic factors, perceived neighborhood walkability also remained significantly associated with engaging in >150 min per week of physical activity (OR = 1.403, *p* = .018). Perceived availability of healthy foods was not associated with meeting physical activity guidelines (> 150 min per week) after adjusting for demographic variables.

**Table 3:** Unadjusted and Adjusted Multinomial Regression Models Examining the Associations between Neighborhood Characteristics and Physical Activity Levels in the Lawrence Health and Well-being Study, 2011–2013

	Model 1			Model 2		
		95 % C.I.			95 % C.I.	
Physical activity level	OR	Lower	Upper	OR	Lower	Upper
>0 and <150 min per Week						
Violence	1.035	0.741	1.446	1.080	0.761	1.533
Safety	0.975	0.766	1.240	0.999	0.817	1.222
Aesthetic Quality	0.962	0.733	1.263	0.867	0.647	1.161
Walking Environment	1.024	0.737	1.422	0.948	0.669	1.344
Availability of Healthy Foods	1.039	0.840	1.284	1.037	0.832	1.291
Social Cohesion	1.120	0.848	1.479	1.003	0.747	1.346
Activities with Neighbors	1.154	0.893	1.492	1.016	0.827	1.401
>150 min Per Week						
Violence	1.178	0.910	1.524	1.128	0.857	1.483
Safety	1.016	0.840	1.229	0.931	0.719	1.204
Aesthetic Quality	0.970	0.782	1.203	0.949	0.753	1.196
Walking Environment	1.445**	1.106	1.888	1.403*	1.061	1.857
Availability of Healthy Foods	1.201*	1.015	1.422	1.150	0.965	1.369
Social Cohesion	1.106	0.890	1.375	1.062	0.839	1.345
Activities with Neighbors	1.159	0.946	1.420	1.140	0.921	1.411

Note. The reference category is: No Activity

Model 1 is unadjusted

Model 2 is adjusted for age, gender, education level, BMI, and smoking status

*p < .05

**p < .01

### The environment and sedentary behavior

Table 4 presents the results of the multiple linear regression models examining the association between neighborhood characteristics, namely perceived neighborhood violence, safety, aesthetic quality, walking environment, availability of healthy foods, social cohesion, and activities with neighbors and sedentary behavior. In the unadjusted analyses, perceived neighborhood violence (β = .115, *p* = .005), safety (β = -.093, *p* = .023), and aesthetic quality (β = -.144, *p* < .001) were associated with sedentary behavior. There were no associations between perceived walking environment, availability of healthy foods, social cohesion, or activities with neighbors and sedentary behavior in the unadjusted analysis. However, after controlling for demographic factors (i.e. age, gender, BMI, smoking), activities with neighbors (β = .072, *p* = .05) was associated with sedentary behavior. The association between neighborhood violence and aesthetic quality with sedentary behavior was no longer significant after adjusting for demographic variables.

**Table 4:** Unadjusted and Adjusted Linear Regression Models Examining the Associations between Neighborhood Characteristics and Sedentary Behavior in the Lawrence Health and Well-being Study, 2011–2013

	Model 1				Model 2			
Neighborhood characteristic	B	SE	β	*p*	B	SE	β	*p*
Violence	0.792	0.282	.115^b^	0.005	0.399	0.265	0.052	0.133
Safety	−0.478	0.211	−0.093^a^	0.023	−0.231	0.199	−0.044	0.247
Aesthetic Quality	−0.835	0.237	-.144^b^	< .001	−0.334	0.226	−0.057	0.14
Walking Environment	−0.500	0.29	−0.071	0.085	−0.271	0.270	−0.038	0.317
Availability of Healthy Foods	−0.080	1.86	−0.018	0.667	−0.086	1.72	−0.019	0.618
Social Cohesion	−0.326	0.241	−0.055	0.177	0.210	0.232	0.035	0.367
Activities with Neighbors	0.183	0.224	0.034	0.413	0.397	0.208	0.072^a^	0.05

Note: B indicates unstandardized coefficient, β indicates standardized coefficient

Model 1 is unadjusted

Model 2 is adjusted for age, gender, education level, BMI, and smoking status

^a^Significant at .05 Level

^b^Significant at .01 Level

## Discussion

In this sample of predominantly low SES Latinos in the northeastern United States, engaging in the recommended levels of physical activity (>150 min per week) was associated with neighborhood walkability. Latinos who lived in more walkable neighborhoods were more likely to meet physical activity guidelines. Conversely, physical features (i.e., walkability, aesthetic quality, and safety) of the neighborhood environment were not related to sedentary behavior in this sample, and sedentary behavior was related to greater engagement in activities with neighbors.

According to frameworks such as social cognitive theory and the social ecological model, behavior has multiple levels of influence including individual, physical and social environment, and organizational levels [33, 34]. As such, individual behaviors are influenced by and also influence the physical or social context in which the behavior takes place [13, 33]. Our study results suggest that environmental influences are behavior-specific and, thus, neighborhood-based interventions for increasing physical activity and reducing sedentary behavior may require different intervention targets.

Our findings are consistent with studies conducted with other populations in the U.S. and abroad that have found neighborhood walkability to be positively associated with physical activity [17–19]. Previous studies of Latinos included only Latina women, thus this study is novel in that it includes a more representative sample of Latino adults. Because this population is at higher risk for diseases preventable by regular physical activity, a better understanding of the environmental correlates of physical activity is critical. For example, Latina women have reported safety, heavy traffic, and poor access to places to exercise as barriers to physical activity [23, 35]. Suggestions to improve the neighborhood walkability could include improvement of sidewalks and ensuring that neighborhoods have safe places for residents to walk [23]. By incorporating the physical environment for promoting healthy behaviors, researchers and public health practitioners may be able to create more effective interventions.

To our knowledge, this was the first study evaluating the relationship between selected neighborhood environment characteristics and sedentary behavior in a sample of male and female Latino adults. An important finding of this study was the lack of relationship between characteristics of the physical environment and sedentary behavior. This is consistent with a review that concluded that, in contrast with its association with physical activity, physical features of the neighborhood environment may be unrelated to sedentary behavior [24]. Our findings do suggests that, given the relationship between sedentary behavior and activities with neighbors, additional research is needed among Latinos to examine how the social environment may influence sedentary behavior. The negative direction of the association between activities with neighbors and sedentary behavior suggests that these social relationships may center on sedentary behaviors. These activities might include sitting while eating dinner, watching television, or simply hanging out. Latinos often come from a collectivist culture where family and social ties are important and common, thus it may be that intervening within these well-established social networks may be effective for reducing sedentary time of individuals as well as their family and social network. It may also be critical to further investigate the culturally-specific nature of different social engagements in order to further understand social norms within networks when evaluating the ability of social neighborhood attributes to reduce sedentary behavior. For, developing interventions that promote culturally-acceptable social engagements that collectively reduce sedentary behavior may be an effective strategy for sedentary behavior change.

A limitation of this study was the cross-sectional design. Future longitudinal research studies are needed to gain additional insight into the long-term and causal effects of neighborhood environments on physical activity and sedentary behaviors of Latinos. Another limitation of this study was the self-report nature of our measures. Measures of the neighborhood environment based on self-report represent perceptions that may or may not fully reflect the reality of people’s neighborhoods. However, the self-report measure used in this study has demonstrated neighborhood reliabilities ranging from 0.76 to 0.88 for neighborhoods defined as census tracts [11], which we believe attenuates this limitation. Furthermore, self-report measures of behavior present biases that over- or under-estimate true physical activity levels or sedentary behavior [36] due to variations in participant recall and interpretation of questions [36]. Additionally, we incorporated a physical activity questionnaire that has been only validated in women [30]. However, we believe that this concern is minimized, as the Women’s Health Initiative (WHI) physical activity questionnaire has been validated against a similar self-report measure, the 7-day Physical Activity Recall (PAR), as well as accelerometers [30, 37]. Furthermore, studies examining these other self-report measures and accelerometers have found no differences in validity by gender [38, 39]. Finally, the study sampling procedures sought to facilitate recruitment of a representative sample of U.S. Latino adults living in the Lawrence, MA area, and most of whom are of Caribbean origin, and thus findings may not be generalizable to other Latino groups.

This study also had several strengths. First, this was the first study examining characteristics of the neighborhood environment associated with physical activity and sedentary behavior in a sample of both male and female Latino adults. Another strength of this study was that we evaluated, separately, physical activity and sedentary behavior. Previous research has often characterized sedentariness as engaging in less than the recommended amount of physical activity per day [9, 40]. Research is now suggesting that physical activity and high sedentariness can co-exist, and each should be viewed as a unique set of behaviors with unique determinants [9]. Finally, this study examined distinct aspects of the neighborhood environment, namely social and physical environmental factors. By identifying social aspects of the neighborhood environment, this study contributes to the existing scientific understanding of the impact of social networks on behavior.

## Conclusion

Both social and physical neighborhood environment factors may influence physical activity and sedentary behavior among Latino adults [34]. Focusing on a greater understanding of the distinct social and physical environmental correlates of physical activity and sedentary behavior, both linked to CVD risk, may provide important insights for reducing CVD risk and health disparities among Latinos, a population with high CVD risk. This study was novel in that we examined differences in neighborhood environment factors associated with physical activity and sedentary behavior among Latinos. Future research establishing longitudinal and causal relationships between selected neighborhood environmental characteristics and both physical activity and sedentary behavior will aid in understanding the acute and long-term effects of neighborhoods on health behavior and disease risk in at-risk populations such as Latinos. Such data are critical for the development of targeted public health interventions that effectively reduce CVD risk among Latinos.
